# Elevated Lipoprotein-Associated Phospholipase A_2_ Independently Affects Age-Related Increases in Systolic Blood Pressure: A Nested Case-Control Study in a Prospective Korean Cohort

**DOI:** 10.1155/2020/5693271

**Published:** 2020-03-31

**Authors:** Hye Jin Yoo, Minjoo Kim, Sang-Hyun Lee, Jong Ho Lee

**Affiliations:** ^1^National Leading Research Laboratory of Clinical Nutrigenetics/Nutrigenomics, Department of Food and Nutrition, College of Human Ecology, Yonsei University, Seoul 03722, Republic of Korea; ^2^Research Center for Silver Science, Institute of Symbiotic Life-TECH, Yonsei University, Seoul 03722, Republic of Korea; ^3^Department of Food and Nutrition, College of Life Science and Nano Technology, Hannam University, Daejeon 34430, Republic of Korea; ^4^Department of Family Practice, National Health Insurance Corporation, Ilsan Hospital, Goyang 10444, Republic of Korea

## Abstract

Inflammatory markers are susceptible to changes over time. Thus, we observed changes in inflammatory markers correlating with age-related increases in blood pressure (BP) through a prospective study. The aim of this study was to investigate changes in inflammatory markers that correlate with age-related increases in BP. The study included 1,500 nondiabetic and normotensive healthy subjects at baseline. Of these, 121 individuals who developed hypertension (defined as systolic BP ≥ 140 mmHg or diastolic BP ≥ 90 mmHg) after 2 years formed the hypertension group. For each incident hypertension case, 2 age- and sex-matched control subjects were selected among those who did not develop hypertension (control group, *n* = 242). After baseline adjustment, the hypertension group exhibited greater increases in body mass index (BMI), systolic and diastolic BP, triglyceride, total cholesterol, glucose, Lp-PLA_2_ activity, and urinary 8-epi-prostaglandin F_2α_ (8-epi-PGF_2*α*_) levels compared to the control group. In the hypertension group, changes in (Δ) systolic BP correlated positively with Δ Lp-PLA_2_ activity, which correlated positively with Δ low-density lipoprotein (LDL−) cholesterol and Δ urinary 8-epi-PGF_2*α*_ levels. Moreover, multiple linear regression revealed baseline systolic BP and Δ Lp-PLA_2_ activity to be independent predictors of Δ systolic BP in the hypertension group. Our results suggest that age-related increases in systolic BP may correlate strongly with elevated Lp-PLA_2_ activity and that Lp-PLA_2_ can be considered a biomarker for systolic BP elevation.

## 1. Introduction

Evidence has emerged to support that inflammation may contribute to hypertension development [1, 2]. Systemic inflammation is associated with the progression of endothelial dysfunction [3], resulting in changes in the structure and function of the endothelium that are often obvious during the early stage of hypertension development [4, 5]. Cross-sectional studies have consistently and significantly shown that inflammatory markers and systolic and diastolic blood pressure (BP) correlate positively [6, 7]. According to prospective studies investigating inflammatory markers, high-sensitivity C-reactive protein (hs-CRP) is related to the risk of developing hypertension [8]. In a nested case-control study, interleukin (IL)-6 was less highly related to the risk of developing hypertension than hs-CRP [9]. However, the mechanisms by which inflammatory markers other than hs-CRP and IL-6 are related to hypertension risk remain unclear. Additionally, the analysis of plasma inflammatory markers that are susceptible to changes over time and measured only once at baseline may have introduced arbitrary misclassifications and resulted in an underestimation of the true relative risks [10]. We are also unaware of prospective studies evaluating the relationship between lipoprotein-associated phospholipase A_2_ (Lp-PLA_2_) activity and hypertension risk.

Therefore, we conducted a nested case-control study to investigate changes in inflammatory markers [hs-CRP, tumor necrosis factor (TNF)-*α*, IL-1*β*, IL-6, and Lp-PLA_2_] with incident hypertension in a mean follow-up 2-year prospective cohort of 1,500 nondiabetic and normotensive healthy subjects aged 20 to 69 years. We also measured urinary excretion of 8-epi-prostaglandin F_2*α*_ (8-epi-PGF_2*α*_), which is a sensitive marker of oxidative stress [11, 12].

## 2. Materials and Methods

### 2.1. Subjects

We performed a nested case-control study with a mean follow-up of 2 years in a prospective cohort that included 1,500 nondiabetic (normal fasting glucose (NFG) < 126 mg/dL) and normotensive (defined as systolic BP < 140 mmHg, diastolic BP < 90 mmHg, and no history of antihypertensive medication use) healthy subjects aged 20 to 69 years who received a biennial medical check-up at the National Health Insurance Corporation Ilsan Hospital in Goyang, Korea (from January 2010 to December 2015). Among the patients, 121 subjects (8.07%) developed hypertension (systolic BP ≥ 140 mmHg or diastolic BP ≥ 90 mmHg) after follow-up without taking any medication. These individuals formed a hypertension group (*n* = 121). For each of the 121 cases of incident hypertension, 2 subjects matched based on age (±1 year) and sex were randomly selected from among the subjects who did not develop hypertension as a control (control group, *n* = 242). The exclusion criteria were as follows: current and/or a history of diseases including hypertension, cardiovascular disease (CVD), diabetes, dyslipidemia, liver disease, renal disease, pancreatitis, or cancer; pregnancy or lactation; and the use of any medications or supplements. The study purpose was fully explained to all participants, and each of them provided written informed consent. The Institutional Review Board of Yonsei University and Ilsan Hospital approved the study protocol, which complied with the Declaration of Helsinki.

### 2.2. Anthropometric Measurements

The body weight (UM0703581; Tanita, Tokyo, Japan) and height (GL-150; G-tech International, Uijeongbu, Korea) of each subject were measured while they wore lightweight clothes with no shoes, and corresponding body mass index (BMI) values were calculated (kg/m^2^). A random-zero sphygmomanometer (HM-1101, Hico Medical Co., Ltd., Chiba, Japan) with appropriate-sized cuffs was used to measure systolic and diastolic BP after at least 20 min of rest in a seated position. BP was assessed three times; as differences among the three measurements of systolic BP were always below 2 mmHg, the BPs were deemed stable. The participants were instructed that they should not smoke or drink alcohol before each BP measurement. The mean BP measurement value was used for statistical analysis.

### 2.3. Blood and Urine Sample Collection

Plasma and serum specimens were obtained from the participants. Specifically, venous blood was collected after at least 12 h of an overnight fast in EDTA-treated and serum tubes (BD Vacutainer; Becton, Dickinson and Company, Franklin Lakes, NJ, USA). The tubes were centrifuged (1,200 rpm, 20 min, 4°C) to separate the plasma and serum, and aliquots were stored at −80°C prior to analysis.

Urine specimens were collected after at least 12 h of an overnight fast in a polyethylene tube containing 1% butylated hydroxytoluene. The tubes were immediately protected from light and stored at −20°C prior to analysis.

### 2.4. Biochemical Assessments

Detailed information has been previously described [13]. Levels of serum fasting lipids (fasting triglyceride, total cholesterol, and high-density lipoprotein (HDL)-cholesterol), glucose, insulin, hs-CRP, and cytokines (TNF-*α*, IL-1*β*, and IL-6), and levels of urinary 8-epi-PGF_2*α*_ were measured using commercial kits. Low-density lipoprotein (LDL)-cholesterol was calculated by the Friedewald formula as follows: LDL-cholesterol = Total cholesterol–[HDL-cholesterol + (Triglyceride/5)]. Homeostatic model assessment (HOMA) of insulin resistance (IR) was assessed based on the values of glucose and insulin. Levels of plasma Lp-PLA_2_ activity were also measured using commercial kits, as previously published elsewhere [14].

### 2.5. Statistical Analysis

SPSS version 23.0 (IBM, Chicago, IL, USA) was utilized for statistical analyses. Skewed variables were transformed to a logarithmic form for normalization. For descriptive purposes, the results are presented as means ± standard error (SE) without logarithmic transformation. Statistical significance was considered at a two-tailed *p*-value less than 0.05. Independent *t*-tests were used to compare continuous variables between the control and hypertension groups. Comparisons of nominal variables between the two groups were performed using a Chi-squared test. General linear model analysis was also conducted with adjustment for potential confounding factors. Paired *t*-tests were conducted to verify differences between the values at baseline and 2-year follow-up in each group. Multiple linear regression was applied to verify significant independent predictors of changes in systolic and diastolic BP. Pearson's correlation coefficients were calculated to determine associations between variables. A heat map was produced to visualize and evaluate associations among variables in the study population (MeV v.4.9.0; http://mev.tm4.org).

## 3. Results

### 3.1. Clinical Characteristics and Inflammatory Markers at Baseline and 2-Year Follow-Up

No significant differences were found between the control (normal BP at both baseline and follow-up) and hypertension (normal BP at baseline but development of hypertension during follow-up) groups with regard to baseline characteristics, including sex (51.2% males and 48.8% females), age (controls, 48.7 ± 0.65 years; hypertension group, 49.3 ± 0.97 years), smoking status (controls, 20.2% current smokers; hypertension group, 13.2% current smokers), and alcohol consumption (controls, 59.1% current drinkers; hypertension group, 65.3% current drinkers). As shown in Table 1, subjects who developed hypertension were significantly heavier and had higher systolic and diastolic BP, hs-CRP levels, and IL-1*β* levels at baseline than did subjects who did not develop hypertension during the mean follow-up of 2 years. After 2 years, the hypertension group showed significant increases in BMI, systolic and diastolic BP, total and LDL-cholesterol levels, glucose levels, and HOMA-IR index; these increases were significantly greater than the increases observed in the controls (with the exception of LDL-cholesterol levels and HOMA-IR index) after adjusting for baseline values. Meanwhile, after 2 years, the controls displayed a significant increase in LDL-cholesterol levels. At the 2-year follow-up, higher systolic and diastolic BP, and IL-1*β* levels were found in the hypertension group compared to the controls after adjusting for follow-up BMI (Table 1).

### 3.2. Lp-PLA_2_ Activity and 8-Epi-PGF_2*α*_ at Baseline and 2-Year Follow-Up

**Figure 1:**
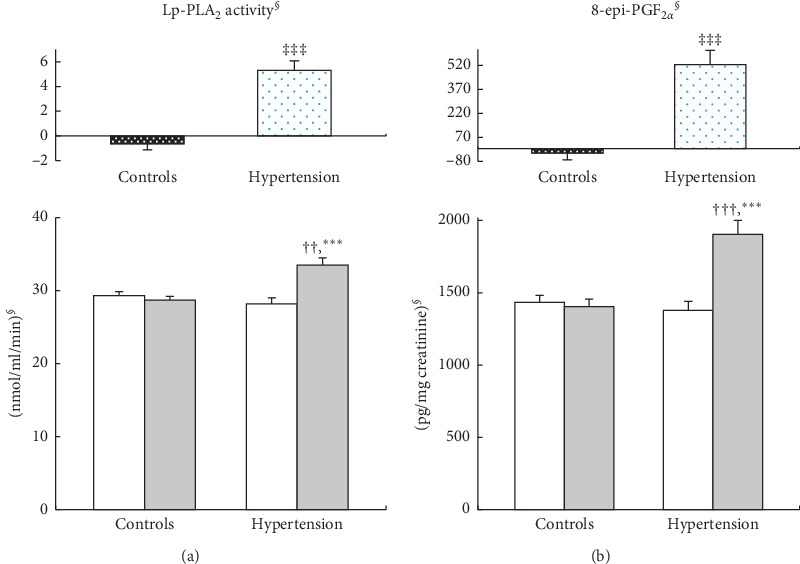
Lp-PLA_2_ activity (a) and 8-epi-PGF_2α_ (b) levels at baseline (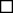) and 2-year follow-up (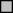) in control (normal BP at both baseline and follow-up) and hypertension (normal BP at baseline but development of hypertension during follow-up) groups. Mean  ±  SE. ^*∮*^tested by logarithmic transformation. ^†^*p* < 0.05,^††^*p* < 0.01,^†††^*p* < 0.001 derived from an independent *t*-test between the groups at baseline and follow-up and adjusted for the baseline BMI and follow-up BMI, respectively. ^*∗*^*p* < 0.05,^*∗∗*^*p* < 0.01,^*∗∗∗*^*p* < 0.001 derived from a paired *t*-test in each group. ^‡^*p* < 0.05,^‡‡^*p* < 0.01,^‡‡‡^*p* < 0.0011 derived from an independent *t*-test at the changed value and adjusted for the baseline values.

No significant differences were observed between the control and hypertension groups in baseline levels of plasma Lp-PLA_2_ activity and urinary 8-epi-PGF_2*α*_ (Figure 1). After 2 years, levels of plasma Lp-PLA_2_ activity and urinary 8-epi-PGF_2*α*_ were significantly increased in the hypertension group; these increases were significantly greater than those in controls after adjustment for the baseline values. At the 2-year follow-up, the hypertension group exhibited higher Lp-PLA_2_ activity and 8-epi-PGF_2*α*_ levels than the control group after adjusting for follow-up BMI (Figure 1).

### 3.3. Correlations between Changes in Clinical Variables, Inflammatory Markers, and Oxidative Stress Markers

**Figure 2:**
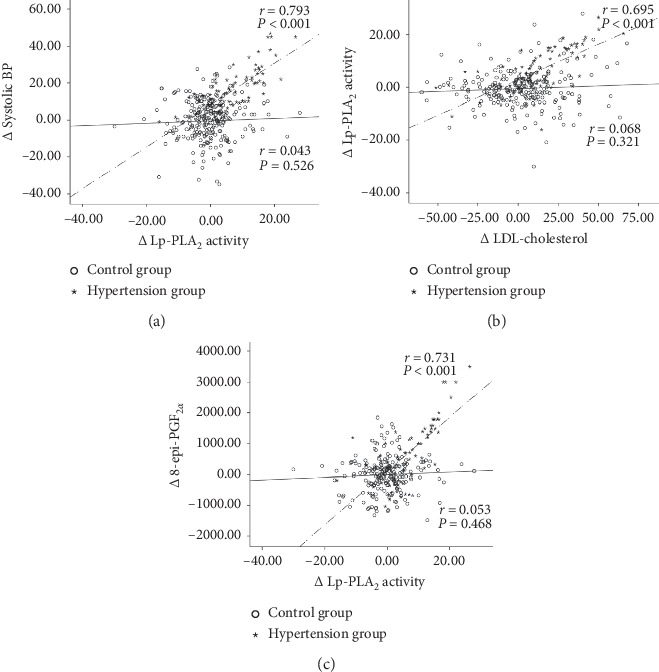
Relationships of the changes in (Δ) Lp-PLA_2_ activity with Δ systolic BP, Δ LDL-cholesterol, and Δ 8-epi-PGF_2α_ in control (normal BP at both baseline and follow-up, solid line) and hypertension (normal BP at baseline but development of hypertension during follow-up, broken line) groups. (a) Correlation between Δ Lp-PLA_2_ activity and Δ systolic BP. (b) Correlation between Δ LDL-cholesterol and Δ Lp-PLA2 activity. (c) Correlation between Δ Lp-PLA_2_ activity and Δ 8-epi-PGF_2α_.

**Figure 3:**
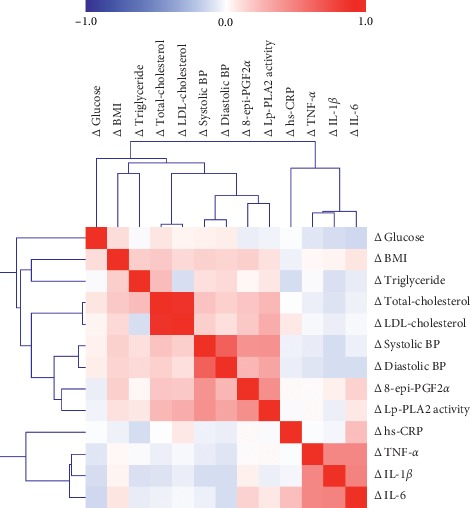
Correlation matrix of associations among changes in (Δ) clinical parameters, inflammatory markers, and oxidative stress markers in all study subjects. For all study subjects (*n* = 363), Pearson's correlation coefficients were calculated to determine the association between variables: changes in (difference from baseline, Δ) clinical characteristics, biochemical parameters, inflammatory markers, and oxidative stress markers. *Red* indicates a positive correlation; *blue* indicates a negative correlation. In particular, Δ Lp-PLA_2_ activity showed strong positive correlations with Δ systolic BP, Δ diastolic BP, and Δ 8-epi-PGF_2α_ (*r* = 0.418, *p* < 0.001; *r* = 0.329, *p* < 0.001; and *r* = 0.430, *p* < 0.001, respectively).

Figure 2 shows the relationships between changes in (differences from baseline, Δ) Lp-PLA_2_ activity with Δ systolic BP, Δ LDL-cholesterol levels, and Δ urinary 8-epi-PGF_2*α*_ levels in both groups. In the hypertension group, Δ Lp-PLA_2_ activity correlated positively and strongly with Δ systolic BP (*r* = 0.793, *p* < 0.001), Δ LDL-cholesterol (*r* = 0.695, *p* < 0.001) and Δ urinary 8-epi-PGF_2*α*_ (*r* = 0.731, *p* < 0.001) (Figure 2). A correlation matrix of changes in major clinical characteristics, biochemical parameters, and inflammatory markers in all subjects (*n* = 363) is depicted in Figure 3.

Multiple linear regression was conducted within the hypertension group to determine the major clinical factors related to Δ systolic BP or Δ diastolic BP (dependent variables). For Δ systolic BP, baseline values for systolic BP, BMI, hs-CRP, and IL-1*β* and Δ BMI, Δ triglyceride, Δ total cholesterol, Δ glucose, and Δ Lp-PLA_2_ activity were defined as the independent variables. For Δ diastolic BP, baseline values for diastolic BP, BMI, hs-CRP, and IL-1*β* and Δ BMI, Δ triglyceride, Δ total cholesterol, Δ glucose, and Δ Lp-PLA_2_ activity were defined as the independent variables. Baseline systolic BP (standardized *β* = −0.286, *p*=0.005) and Δ Lp-PLA_2_ activity (standardized *β* = 0.604, *p* < 0.001) emerged as independent predictors of Δ systolic BP. The baseline diastolic BP emerged as an independent predictor of Δ diastolic BP (standardized *β* = −0.461, *p*=0.001).

## 4. Discussion

In this study, the hypertension group (normal BP at baseline but development of hypertension during follow-up) experienced an 11.7% increase in systolic BP and a 13.6% increase in diastolic BP during the 2-year period. We investigated age-related alterations in inflammatory markers (hs-CRP, Lp-PLA_2_ activity, TNF-*α*, IL-1*β*, and IL-6) and found that only Δ Lp-PLA_2_ activity was independently associated with age-related increases in systolic BP. This result is consistent with a recent report of prehypertension-associated elevation in circulating Lp-PLA_2_ activity [15]. The results suggest that age-related increases in systolic BP correlate strongly with elevated Lp-PLA_2_ activity.

Prospective studies regarding inflammatory markers and the risk of hypertension have shown contradictory results. For example, hs-CRP has been associated with hypertension risk after adjustment for abdominal obesity in 2 cohorts of middle-aged men and women [16, 17]. In contrast, no association was observed between hs-CRP and IL-6 in age- and sex-adjusted analyses of 795 initially normotensive diabetic men and women [18]. Recently, elevated plasma inflammatory markers, including hs-CRP and IL-6, have been reported to be nonsignificantly related to a higher risk of hypertension [10]. In the present study, baseline hs-CRP and IL-1*β* levels in the hypertension group were significantly higher than those in the control group (normal BP at both baseline and follow-up), whereas baseline IL-6 levels showed an increasing trend. However, Δ hs-CRP, Δ TNF-*α*, Δ IL-1*β*, and Δ IL-6 levels were not significantly different between the groups. Indeed, only Δ Lp-PLA_2_ activity was substantially higher in the hypertension group than in the control group. This report is the first prospective study to evaluate the association between Δ Lp-PLA_2_ activity and Δ BP.

The hypertension group exhibited an 18.8% increase in Lp-PLA_2_ activity for the 2-year period, whereas significant alteration in Lp-PLA_2_ activity did not occur in the control group. Lp-PLA_2_ is primarily generated by macrophages but is also produced by monocytes, T lymphocytes, mast cells, and hepatic cells [19, 20]. In the bloodstream, Lp-PLA_2_ primarily circulates bound to LDL-cholesterol [20]. The current study also showed a strong positive correlation between Δ LDL-cholesterol levels and Δ Lp-PLA_2_ activity in the hypertension group. Elevated Lp-PLA_2_ activity has been demonstrated to be associated with endothelial dysfunction, and endothelial dysfunction caused by Lp-PLA_2_ activity plays an important role in BP [21, 22]. Similarly, the hypertension group in this study exhibited significant increases in both Lp-PLA_2_ activity and BP, with a strong positive correlation between Δ Lp-PLA_2_ activity and Δ systolic BP.

Increases in Lp-PLA_2_ activity in the hypertension group occurred in conjunction with enhanced urinary excretion of 8-epi-PGF_2*α*_, which is a sensitive marker of oxidative stress [11, 12]. Lp-PLA_2_, which is bound to LDL-cholesterol, hydrolyzes the ester bond at the *sn*-2 position and generates atherogenic byproducts (oxidized free fatty acids and lysophosphatidylcholines) [23, 24]. Stafforini et al. [23] demonstrated that secreted Lp-PLA_2_ produces F_2_-isoprostanes from the phosphatidylcholine at the *sn*-2 position with high affinity. Additionally, intracellular Lp-PLA_2_, which shows homology with plasma Lp-PLA_2_, has been reported to be involved in the metabolism of esterified 8-epi-PGF_2*α*_ [25]. In the current study, a positive relationship between Δ Lp-PLA_2_ activity and Δ8-epi-PGF_2*α*_ levels was observed for the hypertension group. The age-related increases in Lp-PLA_2_ activity in the hypertension group may be, at least partially, explained by the higher level of oxidative stress compared with the controls at the 2-year follow-up.

Additionally, the hypertension group showed higher mean changes in fasting glucose than the controls. However, no correlations were found between Δ BP and Δ fasting glucose or between Δ BP and Δ HOMA-IR index. These results suggest that elevated Lp-PLA_2_ activity precedes changes in insulin resistance during a span of elevated systolic BP and occurs in conjunction with progressed oxidative stress. Accordingly, this observation certainly indicates that individuals with newly diagnosed mild hypertension should be given appropriate lifestyle advice, including dietary recommendations to reduce LDL-cholesterol, Lp-PLA_2_ activity, and oxidative stress, to reduce the risk of cardiovascular disease.

The limitation of this study was its small sample size. Additionally, the generalizability of the observed associations with Lp-PLA_2_ activity and hypertension to other populations with different ages, ethnicities, and socioeconomic statuses is unknown. Finally, we could not demonstrate why Lp-PLA_2_ activity only showed a correlation with an increase in systolic BP but not in diastolic BP. In addition to our study, several others have reported a positive association between Lp-PLA_2_ activity and systolic BP [26–28]. To the best of our knowledge, the underlying mechanisms that explain why only systolic BP mostly correlates with Lp-PLA_2_ activity have not been elucidated. Therefore, attempts to verify the relationship between systolic BP and Lp-PLA_2_ activity and between diastolic BP and Lp-PLA_2_ activity are needed in the future.

Despite these limitations, the present study demonstrates that age-related increases in systolic BP correlate strongly with elevated Lp-PLA_2_ activity. This suggests that Lp-PLA_2_ should be carefully observed to prevent future risk of BP elevation.

## Figures and Tables

**Table 1:** Clinical characteristics of hypertension controls and cases at baseline and follow-up.

	Controls (*n* = 242)		Hypertension (*n* = 121)		*p* ^*a*^	*p* ^*b*^	*p* ^*c*^
	Baseline	Follow-up	Baseline	Follow-up			
BMI (kg/m^2^)	23.7 ± 0.18	23.8 ± 0.18	24.9 ± 0.26	25.2 ± 0.25^*∗∗∗*^	—	—	
Change	0.06 ± 0.05		0.33 ± 0.08				0.001
Systolic BP (mmHg)	116.8 ± 0.71	116.3 ± 0.70	126.0 ± 0.86	140.8 ± 0.82^*∗∗∗*^	<0.001	<0.001	
Change	−0.52 ± 0.73		14.8 ± 1.05				<0.001
Diastolic BP (mmHg)	73.8 ± 0.54	72.9 ± 0.55	79.9 ± 0.66	90.8 ± 0.65^*∗∗∗*^	<0.001	<0.001	
Change	−0.87 ± 0.54		10.9 ± 0.63				<0.001
Triglyceride (mg/dL)^*∮*^	123.9 ± 4.90	122.1 ± 4.96	131.5 ± 6.43	148.6 ± 10.4	0.754	0.120	
Change	−1.83 ± 3.90		17.2 ± 7.61				0.007
Total cholesterol (mg/dL)^*∮*^	197.4 ± 2.11	199.2 ± 2.12	197.2 ± 3.04	206.0 ± 3.28^*∗∗∗*^	0.470	0.117	
Change	1.81 ± 1.62		8.83 ± 2.40				0.010
HDL-cholesterol (mg/dL)^*∮*^	52.5 ± 0.85	51.9 ± 0.90	51.6 ± 1.14	51.1 ± 1.07	0.609	0.307	
Change	−0.60 ± 0.65		−0.55 ± 0.86				0.856
LDL-cholesterol (mg/dL)^*∮*^	120.8 ± 2.00	123.5 ± 1.82^*∗*^	120.5 ± 2.78	127.3 ± 2.89^*∗∗*^	0.428	0.570	
Change	2.72 ± 1.53		6.78 ± 2.10				0.094
Glucose (mg/dL)^*∮*^	91.4 ± 0.66	91.8 ± 0.65	92.6 ± 1.12	95.2 ± 1.25^*∗∗*^	0.757	0.057	
Change	0.42 ± 0.59		2.66 ± 0.94				0.008
Insulin (*μ*IU/dL)^*∮*^	9.05 ± 0.40	8.87 ± 0.44	8.48 ± 0.31	9.42 ± 0.43	0.096	0.925	
Change	−0.17 ± 0.54		0.94 ± 0.39				0.302
HOMA-IR^*∮*^	2.06 ± 0.11	2.03 ± 0.10	1.92 ± 0.07	2.21 ± 0.11^*∗*^	0.100	0.672	
Change	−0.03 ± 0.14		0.29 ± 0.11				0.208
hs-CRP (mg/dL)^*∮*^	1.00 ± 0.11	1.24 ± 0.22	1.48 ± 0.23	1.32 ± 0.21	0.013	0.985	
Change	0.23 ± 0.24		−0.15 ± 0.30				0.889
TNF-*α* (pg/mL)^*∮*^	9.83 ± 0.74	10.2 ± 1.22	11.5 ± 1.37	10.7 ± 0.92	0.487	0.067	
Change	0.39 ± 0.92		−0.78 ± 1.57				0.726
IL-1*β* (pg/mL)^*∮*^	0.73 ± 0.06	0.74 ± 0.07	0.91 ± 0.10	0.86 ± 0.06	0.028	0.003	
Change	0.01 ± 0.04		−0.05 ± 0.10				0.969
IL-6 (pg/mL)^*∮*^	3.64 ± 0.25	3.61 ± 0.23	4.10 ± 0.34	3.92 ± 0.36	0.086	0.420	
Change	−0.03 ± 0.22		−0.18 ± 0.43				0.806

Mean ± SE. ^*∮*^tested by logarithmic transformation. *p*^*a*^-values derived from an independent *t*-test and adjusted for BMI at baseline. *p*^*b*^-values derived from an independent *t*-test and adjusted for BMI at follow-up. *p*^*c*^-values derived from an independent *t*-test and adjusted for baseline values at the changed value. ^*∗*^*p* < 0.05,^*∗∗*^*p* < 0.01,  and ^*∗∗∗*^*p* < 0.001 are derived from a paired *t*-test.

## Data Availability

The data used to support the findings of this study are available from the corresponding author upon request.
